# Sestrin2 Mediates Metformin Rescued the Age-Related Cardiac Dysfunctions of Cardiorenal Syndrome Type 3

**DOI:** 10.3390/cells12060845

**Published:** 2023-03-08

**Authors:** Migdalia Iglesias, Hao Wang, Meredith Krause-Hauch, Di Ren, Linda Ines Zoungrana, Zehui Li, Jie Zhang, Jin Wei, Nikita Yadav, Kshama Patel, Mohammad Kasim Fatmi, Ruisheng Liu, Edward J. Lesnefsky, Ji Li

**Affiliations:** 1 Department of Surgery, Morsani College of Medicine, University of South Florida, Tampa, FL 33620, USA; 2 James A. Haley Veterans’ Hospital, Tampa, FL 33612, USA; 3 Department of Molecular Pharmacology & Physiology, Morsani College of Medicine, University of South Florida, Tampa, FL 33620, USA; 4 Pauley Heart Center, Division of Cardiology, Department of Internal Medicine, Virginia Commonwealth University, Richmond, VA 23284, USA; 5 Cardiology Section, Medical Service, Richmond Department of Veterans Affairs Medical Center, Richmond, VA 23249, USA

**Keywords:** cardiorenal syndrome 3, acute kidney injury, Sestrin2, metformin, aging

## Abstract

Acute kidney injury (AKI) leads to acute cardiac injury and dysfunction in cardiorenal syndrome Type 3 (CRS3) through oxidative stress (OS). The stress-inducible Sestrin2 (Sesn2) protein reduces reactive oxygen species (ROS) accumulation and activates AMP-dependent protein kinase (AMPK) to regulate cellular metabolism and energetics during OS. Sesn2 levels and its protective effects decline in the aged heart. Antidiabetic drug metformin upregulates Sesn2 levels in response to ischemia–reperfusion (IR) stress. However, the role of metformin in CRS3 remains unknown. This study seeks to explore how the age-related decrease in cardiac Sesn2 levels contributes to cardiac intolerance to AKI-induced insults, and how metformin ameliorates CRS3 through Sesn2. Young (3–5 months) and aged (21–23 months) C57BL/6J wild-type mice along with cardiomyocyte-specific knockout (cSesn2*^−/^^−^*) and their wild type of littermate (Sesn2*^f/f^*) C57BL/6J mice were subjected to AKI for 15 min followed by 24 h of reperfusion. Cardiac and mitochondrial functions were evaluated through echocardiograms and seahorse mitochondria respirational analysis. Renal and cardiac tissue was collected for histological analysis and immunoblotting. The results indicate that metformin could significantly rescue AKI-induced cardiac dysfunction and injury *via* Sesn2 through an improvement in systolic and diastolic function, fibrotic and cellular damage, and mitochondrial function in young, Sesn2*^f/f^*, and especially aged mice. Metformin significantly increased Sesn2 expression under AKI stress in the aged left-ventricular tissue. Thus, this study suggests that Sesn2 mediates the cardioprotective effects of metformin during post-AKI.

## 1. Introduction

Acute kidney injury (AKI) consists of an abrupt decrease in or cessation of kidney function, characterized by increased levels of plasma creatinine (PCr) [1]. AKI occurs in approximately 20% of adults and 33% of children hospitalized with acute illness, and 67% of ICU patients [1,2]. Cardiorenal syndrome Type 3 (CRS3) is a subtype of cardiorenal syndrome (CRS) in which AKI leads to acute cardiac injury and dysfunction. AKI can either directly or indirectly lead to cardiac abnormalities in CRS3 [1]. The direct mechanisms through which AKI interacts with the heart include cellular apoptosis, the activation of the sympathetic nervous and renin–angiotensin–aldosterone systems, and oxidative stress [3]. Direct effects on cardiac function can consist of alterations in cardiomyocyte contractility, myocardial infarctions, and vasoconstriction [4], while indirect mechanisms include electrolyte imbalance, and the accumulation of fluid and uremic toxins [3]. The indirect cardiac effects of AKI can be hypertension, arrhythmias, and pericarditis [4].

Oxidative stress (OS) is the imbalance between the amount of reactive oxygen species (ROS) and antioxidants. OS can damage DNA, lipids, and proteins, leading to conditions such as cancer, and neurodegenerative and cardiovascular diseases [5,6]. Renal ischemia-reperfusion injury (IRI) is a common way via which AKI is induced, and is a risk factor for the development of CRS3 [5]. Renal IRI leads to ROS accumulation in both the kidneys and heart, which promotes cellular damage. Cellular and tissue damage induces an inflammatory response. Increased levels of proinflammatory factors such as tumor necrosis factors (TNFs) and interleukins (ILs) are found in the heart after renal IRI and during AKI [3]. Persistent exposure to these proinflammatory factors can incite cardiac functional and structural abnormalities, such as reduced fractional shortening, increased collagen volume fraction, and the presence of fibrosis [3,7]. These abnormalities are exacerbated by aging. Aging is a risk factor for CRS3, and is associated with ROS accumulation and low levels of cardiac Sestrin2 (Sesn2) [8].

Sesn2 is a stress-inducible protein that acts as a metabolic regulator by activating AMP-dependent protein kinase (AMPK) that, in turn, inhibits the mammalian target of rapamycin complex 1 (mTORC1) [9]. Sesn2 also displays antioxidant properties by reducing ROS accumulation, and providing cardioprotective effects against oxidative and ischemia–reperfusion (IR) stresses. Additionally, Sesn2 can repress cardiac proinflammatory signals through the downregulation of IL-17 signaling cascades [9]. Antidiabetic drug metformin contributes to the upregulation of Sesn2. Metformin can decrease ATP concentration in a cell through the AMPK-activated downregulation of mTORC1, leading to Sesn2 upregulation in response to energetic and ischemia–reperfusion (IR) stress [7]. During IR stress, metformin attenuates left-ventricular (LV) and mitochondrial dysfunction, inflammatory response, and ROS accumulation [10].

As discussed above, although the cardioprotective effects of Sesn2 and its upregulation through metformin administration in IRI have been reported, these effects in IR-induced AKI are not well-studied. This study hypothesizes that the aging-related decrease in cardiac Sesn2 renders the aged heart more vulnerable to injury and dysfunction following IR-induced AKI, and that metformin can help in ameliorating such cardiac stress via Sesn2.

## 2. Materials and Methods

### 2.1. Experimental Animals

Young (3–5 months) C57BL/6J wild-type mice were purchased from The Jackson Laboratory (Bar Harbor, ME, USA). Aged (21–23 months) C57BL/6J wild-type mice were provided by the National Institute of Aging. Sesn2*^f/f^* mice with a C57BL/6J background were bred in our laboratory. Mice with a cardiomyocyte-specific knockout of Sestrin2 (cSesn2*^−/^^−^*) were generated in our laboratory from the breeding of Sesn2*^f/f^* mice and transgenic Cre mice (purchased from The Jackson Laboratory). Transgenic mice have an autosomally integrated Cre gene driven by the cardiac-specific alpha-myosin heavy chain promoter (αMHC). This animal protocol was approved by the Institutional Animal Care and Use Committee of the University of South Florida.

### 2.2. AKI Surgery and Sample Collection

**Figure 1:**
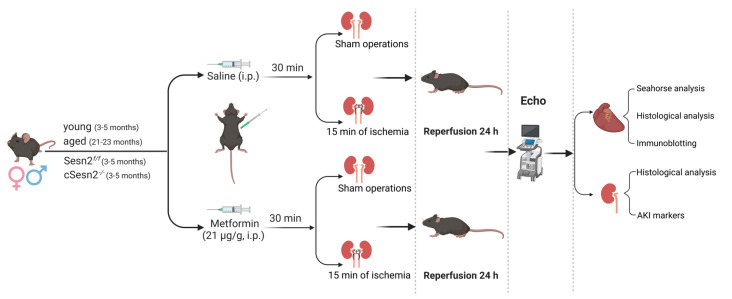
Experimental design. Male and female young and aged C57BL/6J wild-type mice along with cSesn2*^−/^^−^* and Sesn2*^f/f^* C57BL/6J mice underwent a laparotomy and the clamping of the renal artery and vein for 15 min, followed by 24 h of reperfusion. Echocardiograms were performed, and tissues were collected for immunoblotting, histopathological, AKI induction, and mitochondrial respiration analyses.

Male and female mice were randomly assigned to the sham, sham with metformin, AKI, and AKI with metformin groups. Metformin and vehicle group mice received a 21 µg/g metformin intraperitoneal (IP) injection and saline IP injection, respectively, 30 min before surgery. Mice were anesthetized with 1.5% isoflurane and underwent a laparotomy followed by either a sham surgery or AKI surgery. AKI surgery consisted of clamping both renal arteries and veins for 15 min, followed by 24 h of reperfusion. The mice were then anesthetized as described above and euthanized via the rapid excision of the heart (IACUC # 9408R). The left ventricle (LV) and kidney of all mice were isolated and freeze-clamped in liquid nitrogen for the acquisition of total protein or placed in 4% paraformaldehyde in PBS for histological examination (Figure 1).

### 2.3. Immunoblotting

LV total protein underwent SDS-PAGE and electrotransfer to polyvinylidene difluoride membranes (Millipore, Bedford, MA, USA) [6,9]. Rabbit Sesn2 antibody (Cat #10795-1-AP, Cat #21346-1-AP) from Proteintech^®^ (Chicago, IL, USA) and Ab178518 from Abcam (Waltham, MA), were used following the manufacturer’s protocol. Additionally, rabbit GAPDH (Cat # 2118S) was obtained from Cell Signaling Technology (Danvers, MA, USA). Bands were detected with SuperSignal West Femto (Thermo Fisher Scientific, Waltham, MA, USA) and the ChemiDoc XRS+ Gel Imaging System (Bio-Rad Laboratories, Hercules, CA, USA). Intensity values were quantified and analyzed with Image Lab™ Software and expressed relative to GAPDH.

### 2.4. Mitochondrial Respiration Analysis

The oxygen consumption rate (OCR) levels of isolated cardiomyocytes suspended in DMEM media was measured using the Mitochondrial Stress Test in the Seahorse X96 analyzer. Measurements were obtained under basal condition and with the addition of 2 µL of oligomycin, an ATP synthase inhibitor; 6 µL carbonyl cyanide-p-trifluoromethoxyphenylhydrazone (FCCP), an OXPHOS uncoupler that induces maximal respiration; 3 µL antimycin A, a Complex I inhibitor. The results were graphed with the Seahorse software and normalized to 4000 cells per well.

### 2.5. Echocardiography

After the 24 h reperfusion period, the echocardiograms of all mice were performed using the Vevo 3100 imaging system (VisualSonics Inc., Toronto, ON, Canada) to evaluate cardiac function as previously described [11]. Simpson’s measurements were performed to obtain the systolic function values consisting of an LV ejection fraction (LVEF) and fractional shortening (LVFS) and the diastolic function assessed through the early-to-late ventricular filling velocity ratio (E/A) [12].

### 2.6. Histopathology

Renal and LV tissues fixed in 4% paraformaldehyde in PBS were stained with hematoxylin and eosin (H&E) to assess the cellular morphology. LV tissues were stained with Masson’s Trichrome to evaluate the presence of collagenous tissue. The slides were imaged in a blinded fashion using the Keyence BZ-X710 All-in-One Fluorescence Microscope with 20X magnification. The percent collagen volume fraction (CVF) by area was calculated with Fiji ImageJ Version 2.3.0 (ImageJ Software, Madison, WI, USA).

### 2.7. Kidney Function Assessment

To determine plasma creatinine concentration (PCr), blood samples were collected through the tail vein, and PCr was measured via HPLC at the O’Brien Center Core of the University of Alabama, Birmingham [13,14,15].

### 2.8. Statistical Analysis

Statistical analysis was performed using GraphPad Prism 9 (GraphPad Software, Inc., San Diego, CA). One- or two-way ANOVA was used to determine differences among two or more groups, with *p* < 0.05 deemed statistically significant. Data are expressed as means ± standard error of the mean (SEM).

## 3. Results

### 3.1. Metformin Protects Renal Structure and Function following AKI Surgery

**Figure 2:**
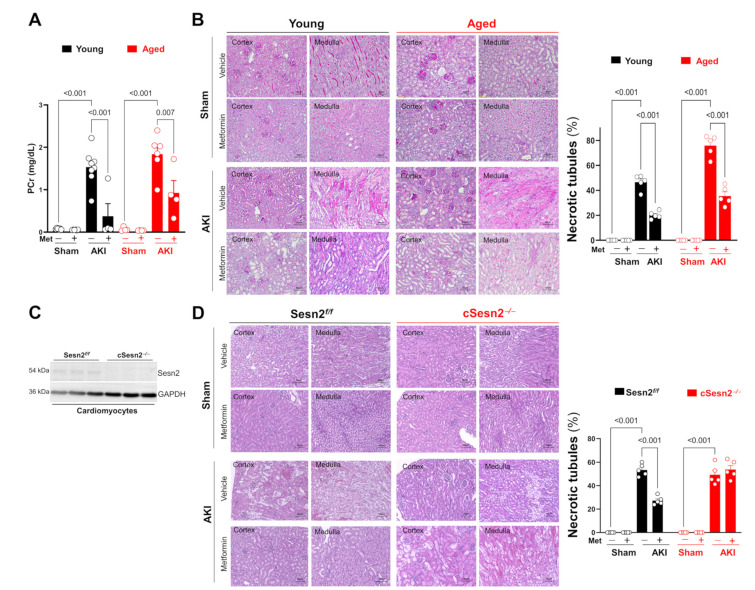
Effects of AKI surgery and metformin on renal structure and function. (**A**) Plasma creatinine concentration (PCr); means ± SEM; *n* = 4–8 per group. (**B**,**D**) Representative H&E staining and statistical analysis; means ± SEM; *n* = 5 per group. (**C**) Immunoblotting of Sesn2 with isolated cardiomyocytes from Sesn2*^f/f^* or cSesn2*^−/^^−^* mouse hearts.

An abrupt change in plasma creatinine concentration (PCr) is a strong indicator of acute kidney injury and dysfunction. The AKI surgery performed in this study significantly increased PCr levels, especially in the aged mice, compared to the sham (Figure 2A). Moreover, metformin was able to significantly reduce PCr levels in both aged and young mice with AKI. The AKI surgery also significantly increased the percentage of necrotic tubules in renal medullae and cortices in all four genotypes, especially in aged mice (Figure 2B–D). Metformin significantly decreased the percentage of necrotic tubules present in all mice after AKI surgery except in cSesn2*^−/^^−^* mice. These results indicate that the IR-AKI surgery successfully induced AKI conditions in all mice, with a more severe effect on the aged mice, and the known protective effects of metformin during IRI could also be seen during IR-AKI.

### 3.2. Metformin Preserves Cardiac Function under AKI Stress

**Figure 3:**
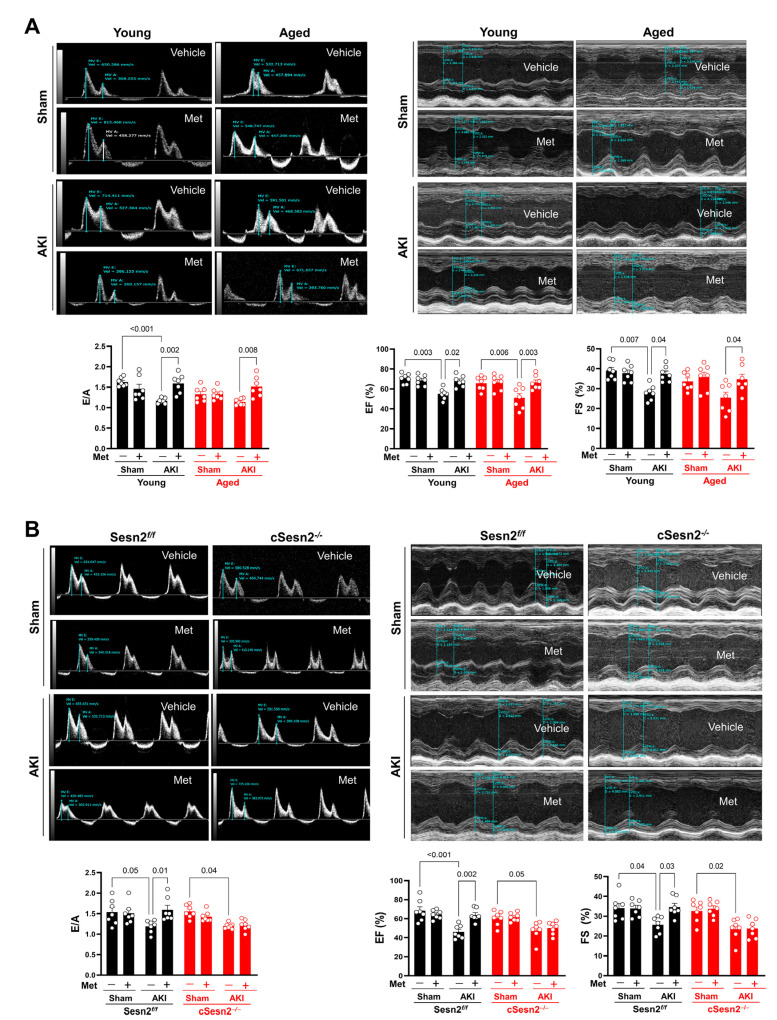
Evaluation of cardiac function through echocardiograms. (**A**) Diastolic function (E/A) and systolic function (LVEF and LVFS) of aged and young mice. (**B**) Diastolic and systolic functions of Sesn2*^f/f^* and cSesn2^−/−^ mice. Means ± SEM; *n* = 7 per group.

Once the successful induction of AKI conditions in the murine kidneys had been confirmed, the effects of CRS3 were evaluated through an assessment of cardiac function. To evaluate the effects of aging on cardiac function following AKI and metformin administration, echocardiograms were performed on young and aged wild-type (WT) mice (Figure 3A). As shown with the E/A ratio, the diastolic function of young WT mice, unlike that of their aged WT littermates, significantly deteriorated under the AKI condition compared to the sham condition. Systolic function, as assessed through LVEF and LVFS, significantly declined in both young and aged WT mice under the AKI condition as compared to the sham. However, a significant decrease in LVFS was not observed in the aged AKI mice when compared to the aged sham mice. Nevertheless, both genotypes showed a significant improvement in post-AKI systolic and diastolic function with metformin administration. To examine metformin’s ability to protect cardiac function via Sesn2, echocardiograms were also performed on Sesn2*^f/f^* and cSesn2*^−/−^* mice (Figure 3B). Although systolic and diastolic function significantly declined in both genotypes under the AKI condition, metformin was only able to significantly rescue systolic and diastolic function in the Sesn2*^f/f^* mice. Such results show that metformin could protect cardiac systolic and diastolic function through Sesn2.

### 3.3. Metformin Preserves Sesn2 Levels in the Heart under AKI Condition

**Figure 4:**
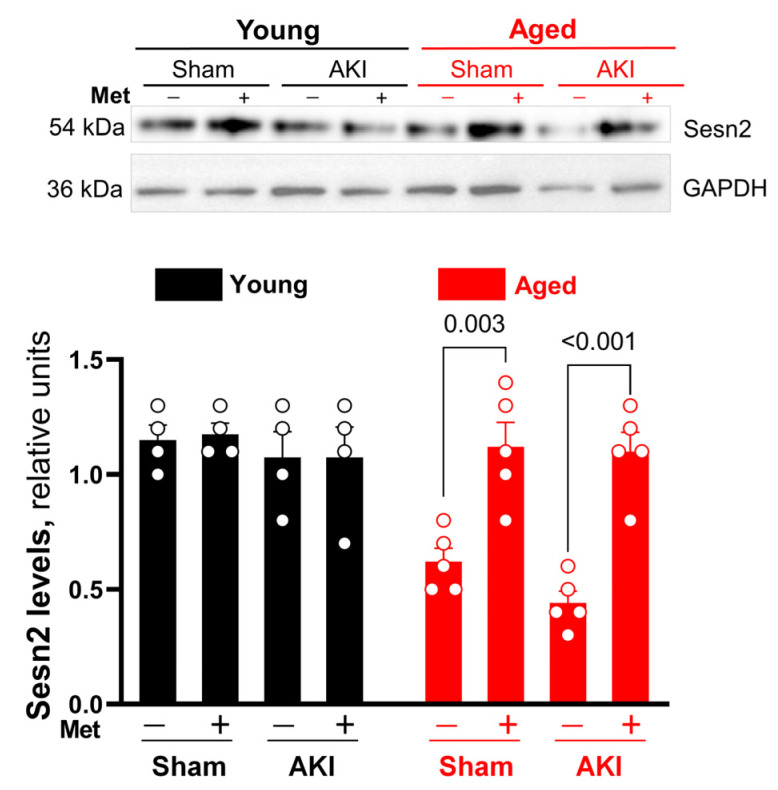
Western blot representative images and statistical analysis. Sesn2 expression in young vs. aged hearts in sham and AKI conditions. Analysis represented as mean ± SEM; *n* = 4–5 per group.

Immunoblotting was performed to assess Sesn2 expression in the left ventricle under the AKI condition and metformin treatment. Data indicated that AKI did not significantly affect Sesn2 expression levels in both the young and the aged group when compared to sham (Figure 4). However, the metformin injection significantly increased Sesn2 levels in aged but not in young hearts under both sham and AKI conditions (Figure 4). These data reveal that metformin administration can rescue impaired cardiac Sesn2 levels under physiological and pathological conditions in aging. However, metformin administration did not have a significant effect on cardiac Sesn2 levels in young mice.

### 3.4. Metformin Provides Protection from Myocardial Cellular and Fibrotic Damage

**Figure 5:**
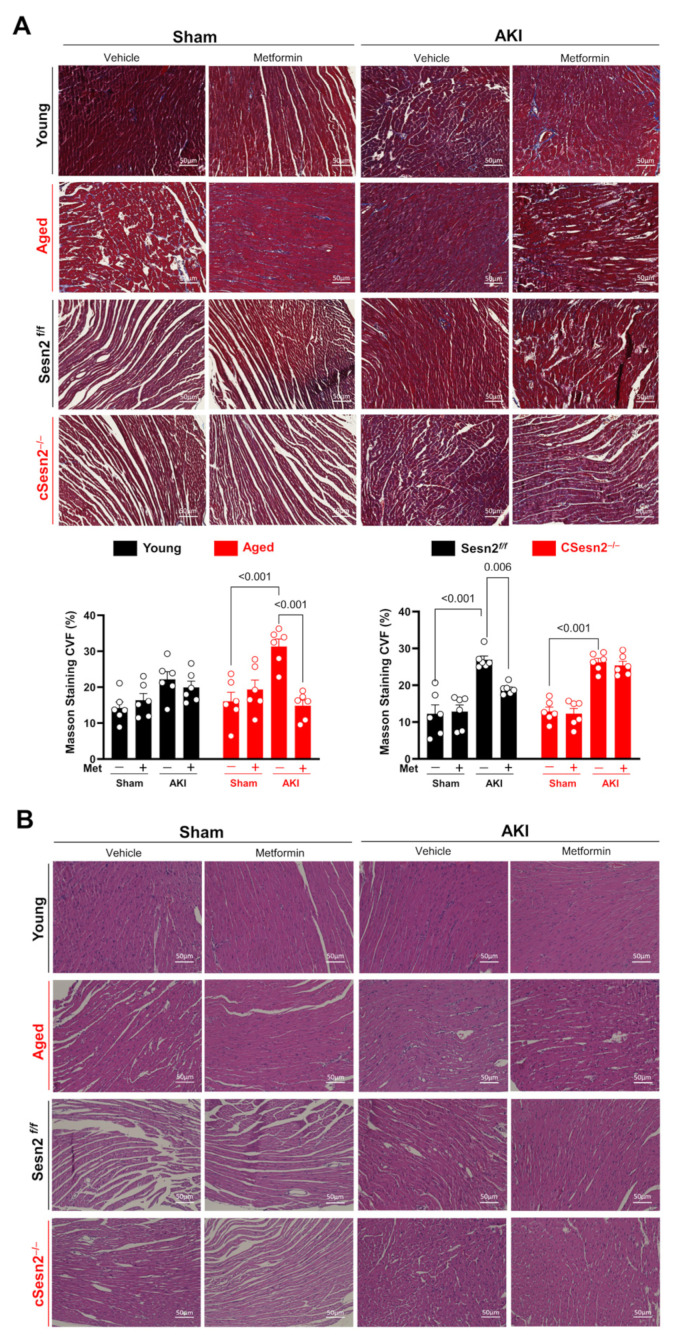
Effects of AKI and metformin on the development of cardiac cellular and fibrotic damage. (**A**) Representative Masson’s trichrome staining and percent collagen volume fraction (CVF). Means ± SEM; *n* = 6 per group. (**B**) Representative hematoxylin and eosin staining.

The accumulation of ROS and inflammatory cytokines does not only affect cardiac function, but also leads to cellular damage and fibrosis. The presence of fibrosis in LV tissue was quantified through the percent collagen volume fraction (CVF) in all mice (Figure 5A). Aged, Sesn2*^f/f^*, and cSesn2^−/−^ mice with AKI had significantly more fibrosis than that of their sham counterparts, with aged mice having the highest average CVF. AKI and metformin did not have a significant effect on the presence of fibrosis in young mice. Meanwhile, metformin significantly reduced fibrosis in the aged and Sesn2*^f/f^* mice with AKI, but not in the cSesn2*^−/^^−^* mice. These results suggest that AKI can lead to the development of myocardial fibrosis, with aged mice being more vulnerable, and that metformin is able to upregulate Sesn2 to save myocardial tissue from such abnormality. The health and integrity of myocardial tissue was further assessed through H&E staining (Figure 5B). Myocardial cells in the aged and cSesn2*^−/^^−^* mice under AKI stress were notably more damaged and experienced a greater loss of their ordered physiological organization as compared to their sham counterparts and to Sesn2*^f/f^* and young LV tissue under AKI stress. Such findings illustrate that the lower levels of Sesn2 in aged and cSesn2*^−/^^−^* mice failed to protect the LV from AKI-induced cellular damage. Despite these lower levels, metformin was still able to noticeably rescue the myocardial cells of both genotypes from AKI-induced damage.

### 3.5. Metformin Preserves Mitochondrial Function in the Heart under AKI Stress

**Figure 6:**
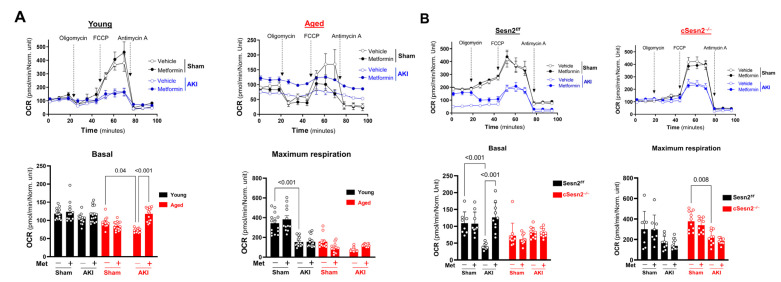
Oxygen consumption rate (OCR) of isolated cardiomyocytes from Seahorse X96 mitochondrial stress test. Mitochondrial stress test profile including Basal and Maximum Respiration of (**A**) young vs. aged mice and (**B**) cSesn2*^−/^^−^* vs. Sesn2*^f/f^* mice. Means ± SEM; *n* = 8–15 per group.

ROS accumulation causes dysfunctions in the electron transport chain (ETC) during IR stress. Therefore, the mitochondrial respiration of isolated cardiomyocytes was measured to assess the effects of IR-AKI and metformin on mitochondrial function. In aged (Figure 6A) and Sesn2*^f/f^* mice (Figure 6B), basal OCR levels significantly decreased after AKI, indicating a cardiomyocyte response to stress conditions and a significant decline in mitochondrial function. This decrease in basal OCR levels could also indicate low ATP demand, and the inhibition of ATP synthase and the ETC overall [16]. These levels significantly increased in the presence of metformin in both genotypes. The basal OCR levels of young (Figure 6A) and cSesn2^−/−^ (Figure 6B) mice were not significantly affected by AKI or metformin administration. In young and cSesn2^−/−^ mice, maximal OCR levels significantly decreased after AKI suggesting deterioration of the structural integrity of the ETC and mitochondria. Metformin did not influence maximal respiration after AKI in any of the four genotypes.

## 4. Discussion

The results of this study suggest that the age-related decline in cardiac Sesn2 levels is attributed to more detrimental cardiac outcomes in CRS3 and that metformin can provide cardiac structural and functional protection against AKI stress through Sesn2. Fifteen minutes of ischemia-induced AKI followed by 24 h of reperfusion deteriorated renal structure and function through the augmentation of the percentage of necrotic tubules and levels of plasma creatinine, both of which were rescued by metformin. Additionally, metformin attenuated systolic and diastolic dysfunction, myocardial cell damage and fibrosis, and the loss of function in the mitochondria of cardiomyocytes in mice affected by AKI stress. These benefits were especially observed in aged mice and absent in cSesn2^−/−^ mice. Overall, this study revealed that metformin could significantly improve cardiac functional and structural integrity during AKI stress via Sesn2.

As previously mentioned, renal IRI induces an inflammatory response in the heart. Elevated levels of cytokines such as ILs and TNFs are associated with indicators of cardiac dysfunction such as a reduction in left ventricular ejection fraction and fractional shortening [1]. The detrimental effects of ischemic-AKI on cardiac systolic function could also be seen in this study through the significant decline in LVEF and LVFS in mice with AKI. While there was not a significant decrease in E/A and LVFS in aged mice with AKI compared to sham, aging alone contributed to a decline in systolic and diastolic function. Therefore, AKI only seemed to make a small contribution to the already existing cardiac dysfunction in aged mice. A study by Jo et al. found that, in rats, metformin can improve left ventricular systolic function after IRI by significantly increasing LVEF and LVFS and diastolic function through an increase in E/E′ ratio [17]. The present study also found that metformin can significantly increase LVEF and LVFS values and diastolic function after IR-AKI stress. The inability of metformin to do so in the cSesn2*^−/−^* mice is likely attributed to the decreased Sesn2 levels in this genotype, meaning that more Sesn2 is likely needed for metformin to provide its protective effects.

The ongoing activation of inflammatory pathways can also incite the development of myocardial fibrosis. Myocardial fibrosis arises due to the excessive production of collagen from fibroblasts caused by aging, injury, and disease [18,19]. IRI leads to the replacement of damaged tissue with fibrotic scars originating from fibroblasts differentiated into myofibroblasts [20,21]. These myofibroblasts overproduce extracellular matrix proteins such as collagen causing fibrosis [21]. Here, a high amount of cardiac fibrotic tissue was found in aged, Sesn2*^f/f^* and cSesn2*^−/−^* mice with AKI as compared to sham conditions. A significant development of fibrosis in the young mice with AKI was not seen, while the aged mice with AKI had the highest CVF. This correlates with previous findings highlighting the increased collagen accumulation and fibrosis in aging animal and human models [22]. Increased fibrosis following IR-AKI was linked to hypertrophic cardiomyopathy and a loss of myocardial elasticity that decreases LVEF [23,24]. Here, a decrease in LVEF was indeed observed in the genotypes affected by fibrosis including aged, Sesn2*^f/f^*, and cSesn2*^−/−^* mice with AKI. Metformin rescues mice from fibrosis and inflammation following IRI [25]. The present study also reports a significant reduction in cardiac fibrotic tissue in IR-AKI with metformin treatment.

AKI generates ROS accumulation in the heart. During ischemia, the electron transport chain (ETC) is in a reduced state; however, during reperfusion, the ETC reacts with oxygen causing the formation of ROS [19]. These ROS species can increase mitochondrial pore permeability, causing mitochondrial dysfunction and in turn more ROS production [26]. Mitochondrial ROS can trigger inflammatory cascades, and lead to cell death and cardiac dysfunction [19]. The mitochondrial respiration analysis in the present study showed that mitochondrial function was indeed impaired with IR-AKI compared to sham conditions. Metformin reduces mitochondrial ROS production by inhibiting reverse electron flow through complex 1 [27]. Metformin also decreases ATP concentration in the cell, which upregulates Sesn2 as a response [28]. Additionally, Ren et al. reported that Sesn2 offers a protective effect against mitochondrial damage in mice hearts after IR stress [29]. Consequently, given the metformin-driven decrease in ROS accumulation and upregulation of Sesn2, mitochondrial function should be significantly rescued by metformin following IRI stress. Indeed, this conclusion held true in the present study, where basal OCR levels were significantly increased in the presence of metformin in aged and Sesn2*^f/f^* mice but not in cSesn2*^−/−^* mice. In the present study, metformin did not influence maximal respiration OCR levels. Although few studies are available on the effects of metformin on the maximal respiration levels of isolated cardiomyocytes, other studies reported its effects on other cell types. Studies by Orang et al., and Geng et al. reported that metformin decreased maximal mitochondrial respiration in colorectal and hepatocellular carcinomas, respectively [30,31].

Immunoblotting results indicate that Sesn2 expression is significantly elevated in both sham and AKI conditions with metformin administration in Sesn2*^f/f^* and aged mice. Under AKI stress, cSesn2*^−/−^*mice also showed significantly elevated Sesn2 expression with metformin administration. Sesn2 knockout in cSesn2^−/−^ mice is also specific only to cardiomyocytes. Therefore, Sesn2 is still present in other myocardial cell types such as endothelial cells, smooth muscle cells, fibroblasts, and immune cells [7]. Consequently, the observed expression and metformin-induced elevation of Sesn2 in cSesn2*^−/−^* tissue can be attributed to this fact. For future studies, performing immunoblotting with mice with a global Sesn2 knockout or with isolated Sesn2-knockout cardiomyocytes could yield different results.

This study demonstrates that aging aggravates the negative effects of AKI on the heart, and that metformin can provide cardioprotective effects during IR-AKI through Sesn2. Furthermore, through AMPK activation, metformin improves LV function, incite anti-inflammatory action, and improve mitochondrial respiration and ATP generation [10]. Therefore, an additional investigation into the Sesn2-AMPK pathway could lead to better comprehension of the mechanism via which Sesn2 offers its cardioprotective effects during CRS3. Furthermore, under OS, Sesn2 is upregulated causing nuclear factor erythroid 2-related factor 2 (Nrf2) to travel from the cytoplasm to the nucleus, where it can offer protection against OS through the expression of Sesn2 [6,8,32]. Given this positive feedback loop, future studies into the connection between Nrf2 and Sesn2 during CRS3 could be beneficial. Additionally, given the importance of inflammatory responses on cardiac systolic and diastolic function and the development of myocardial fibrosis during CRS3, further investigation into the cardiac inflammatory cytokines present during CRS3 and their relationship to Sesn2 could also provide insights into the mechanism by which Sesn2 offers protection. Altogether, this study demonstrates that metformin’s ability to provide cardioprotective effects through Sesn2 could render it a potential target for the treatment of CRS3.

## Data Availability

Not applicable.
